# Microbiome-dependent phytosterol sulfates production and tissue distribution in mice

**DOI:** 10.1016/j.isci.2026.117883

**Published:** 2026-10-16

**Authors:** Koki Tomita, Takeru Nakano, Satoshi Morozumi, Nobuyuki Okahashi, Makoto Arita

**Affiliations:** 1 Division of Physiological Chemistry and Metabolism, Graduate School of Pharmaceutical Sciences, Keio University, 1-5-30 Shibakoen, Minato-ku, Tokyo 105-8512, Japan; 2 Laboratory for Metabolomics, RIKEN Center for Integrative Medical Sciences, 1-7-22 Suehiro-cho, Tsurumi-ku, Yokohama, Kanagawa 230-0045, Japan; 3 Human Biology Microbiome Quantum Research Center (WPI-Bio2Q), Keio University, 35 Shinanomachi, Shinjuku-ku, Tokyo 160-8582, Japan; 4 Department of Bioinformatic Engineering, Graduate School of Information Science and Technology, The University of Osaka, 1-5 Yamadaoka, Suita, Osaka 565-0871, Japan; 5 Cellular and Molecular Epigenetics Laboratory, Graduate School of Medical Life Science, Yokohama City University, 1-7-29 Suehiro-cho, Tsurumi-ku, Yokohama, Kanagawa 230-0045, Japan

**Keywords:** sterol sulfate, sitosterol sulfate, LC-TOF/MS, untargeted lipidomics, bacteria, gut microbiota, bacteroidota, aging

## Abstract

Microbial biotransformation of dietary lipids, such as cholesterol, influences host homeostasis. Despite extensive studies on animal sterols in host-microbiota interactions, the role of phytosterols in homeostasis remains unclear. In this study, we characterized phytosterol sulfates as microbiota-derived metabolites using untargeted lipidomic data mining. The levels of sitosterol sulfate (SIS), campesterol sulfate, and stigmasterol sulfate, the three major phytosterol sulfates, along with coprostanol sulfate, were reduced in the feces of germ-free mice compared with those of specific pathogen-free mice. We characterized a series of Bacteroidota species and their sterol sulfotransferases that produced phytosterol sulfates more efficiently than mammalian enzymes. Furthermore, SIS significantly accumulated in host tissues in a microbiota- and age-dependent manner, whereas unconjugated phytosterols were mostly undetectable. Our work provides insights into host-microbiome interactions via gut bacterial phytosterol metabolism.

## Highlights

• Gut microbiome converts dietary phytosterols into phytosterol sulfates
• *Bacteroides thetaiotaomicron* Sult efficiently produces phytosterol sulfates
• Phytosterol sulfates are distributed across multiple host tissues
• Sitosterol sulfate accumulates in an age-dependent manner

## Graphical abstract

**Figure:**
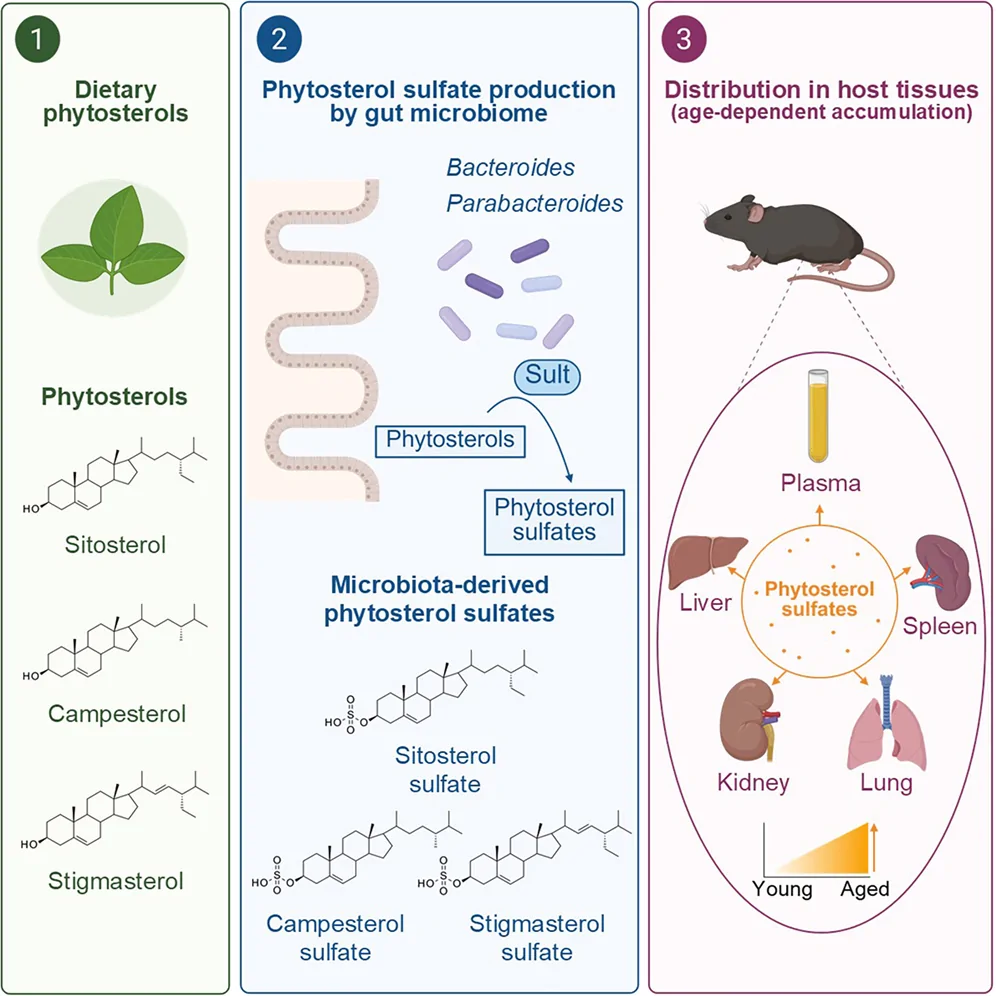

## Teaser

biochemistry; microbiology; microbiome; lipidomics; metabolomics

## Introduction

Cholesterol is an essential lipid that modulates membrane fluidity and stability, and a precursor for bile acids, steroid hormones, and vitamin D.^1^^,^^2^^,^^3^ It is sulfated by host sulfotransferase family 2B member 1 to form cholesterol sulfate (CS), which has immune functions.^4^ CS modulates local immune responses in the skin and eyes by acting as a macrophage-inducible C-type lectin (Mincle) ligand and dedicator of cytokinesis protein 2 (DOCK2) inhibitor.^5^^,^^6^

Phytosterols are characterized by structural variations in the alkyl side chain extending from the C17 position of the steroid backbone.^7^ Their metabolic dynamics substantially differ from those of cholesterol.^8^ Approximately 50% of dietary cholesterol is absorbed, whereas only ∼5% of phytosterols enter the circulation via the intestinal epithelial transporter Niemann-Pick C1-like 1 (NPC1L1).^9^^,^^10^^,^^11^ This is primarily due to the efficient extrusion of phytosterols from enterocytes by the heterodimeric ATP-binding cassette transporters, ABCG5 and ABCG8.^12^ Consequently, phytosterols are present at very low levels in mammalian tissues and do not substantially accumulate.^13^ In humans and other mammals, mutations in the sterol efflux transporters, ABCG5 and ABCG8, lead to the abnormal accumulation of phytosterols and are associated with tendon xanthomas, premature atherosclerosis, thrombocytopenia, and hemolytic anemia.^13^^,^^14^ Therefore, these efflux transporters may play crucial roles in phytosterol levels. These observations suggest that maintaining low phytosterol levels is crucial in animals.^15^^,^^16^ Sterol sulfate levels are also regulated by environmental factors, such as microbiota. Recently, bacterial *sult* homologs were identified in the gut microbiota and shown to metabolize several substrates including hormones, stanols, and plant-derived sterols (phytosterols) to dehydroepiandrosterone sulfate, coprostanol sulfate (CNS), sitosterol sulfate (SIS), and campesterol sulfate (CAS).^17^^,^^18^ Phytosterol sulfates are known as regulatory molecules of a cell death program in *Skeletonema marinoi*, a marine bloom-forming diatom.^19^ Additionally, *in vitro* studies have shown that SIS modulates liposomal membrane fluidity and improves membrane stability.^20^ However, their physiological roles in humans and mice remain largely unknown. Interestingly, although phytosterols themselves are efficiently excluded from host tissues, several microbiota-derived lipids have recently been reported to accumulate in mammalian tissues in an age-dependent manner, highlighting the potential for microbial lipid metabolites to enter and persist within the host.^21^ However, the distribution of phytosterol sulfates and the contribution of microbiota to host sterol sulfate pools remain poorly understood.

Here, through untargeted lipidomics data mining, we identified phytosterol sulfates as gut microbiota-derived metabolites and Bacteroidota-derived Sults as the enzymes responsible for phytosterol sulfate production. Furthermore, we detected phytosterol sulfates in various mouse tissues in a microbiota- and age-dependent manner. Our findings highlight the impact of the gut microbiota on phytosterol metabolic dynamics and provide insights into understanding host-microbiota interactions to maintain sterol homeostasis.

## Results

### Characterization of microbiome-derived sterol sulfates

To examine the bacteria-dependent production of sterol sulfates, we reanalyzed previously published untargeted fecal lipidome data from specific pathogen-free (SPF) and germ-free (GF) mice.^21^ Along with the identification of CS, a series of molecules showing sulfate ion fragments (*m/z* = 96.960) were observed in the MS/MS spectra. These molecules were identified as SIS, CAS, stigmasterol sulfate (STS), and CNS (Figures 1A–1E and S1A–S1E). CS abundance was not significantly reduced in GF mice compared with that in SPF mice (Figure 1A), indicating a minor contribution of the gut microbiota to fecal CS levels in SPF mice. In contrast, the levels of phytosterol sulfates (SIS, STS, and CAS) and CNS significantly decreased in fecal samples from GF mice (Figures 1B–1E). Among these, SIS and STS exhibited the strongest microbiota dependence, with 98.2% and 99.2% reductions in GF mice, respectively, compared with those in SPF mice (Figures 1B and 1D). CAS and CNS abundance were reduced by 87.4% and 64.6%, respectively (Figures 1C and 1E). We next reanalyzed untargeted fecal lipidomic data from mice whose gut microbiota had been artificially eliminated with an antibiotic cocktail (Abx).^22^ CS, SIS, CAS, and STS were consistently detected. In addition, brassicasterol sulfate (BS) was detected in the fecal samples (Figures S2A–S2G). SIS, CAS, STS, and CNS were also significantly reduced in Abx-treated mouse feces compared with that in control mouse feces (Figures S2B–S2D and S2F). These results suggested that some phytosterol sulfates were produced by the microbiota, with SIS being the most prominent.

**Figure 1:**
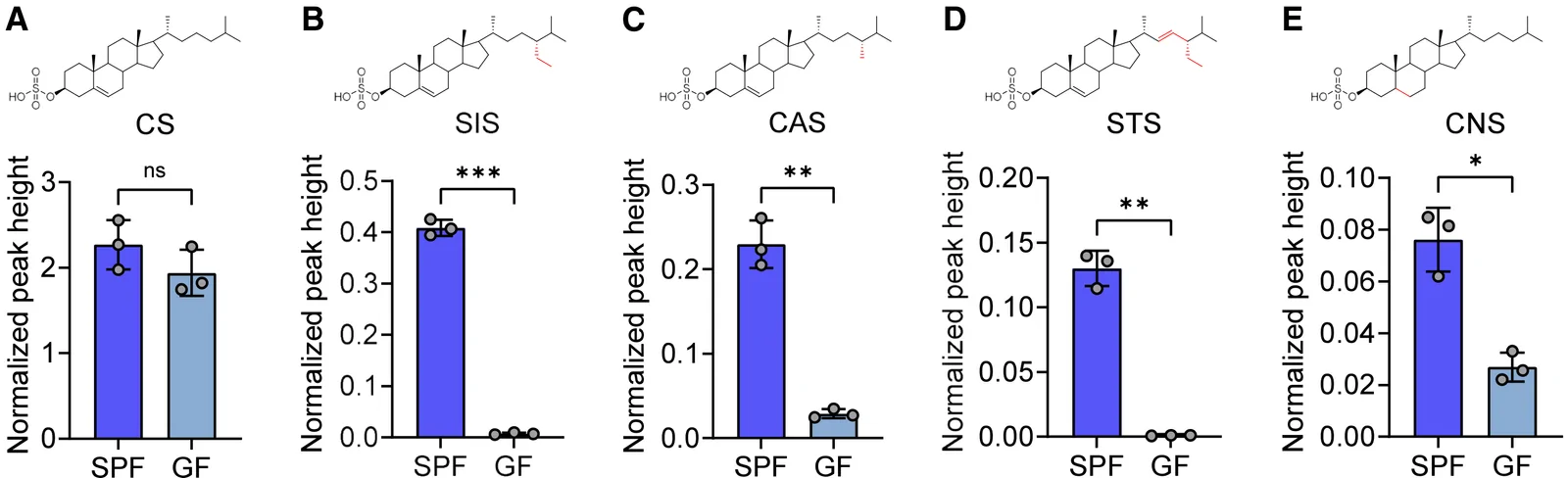
Several sterol sulfates are produced in a bacteria-dependent manner (A–E) The normalized intensities of sterol sulfates in the 2-month-old SPF mice feces vs. GF mice feces. Normalization was performed by dividing the raw intensities of sterol sulfates by the raw intensity of d7-lysophosphatidylcholine added as an internal standard. Bars represent the mean, and error bars indicate SD. Each data point represents an individual mouse (*n* = 3 per group). ∗*p* < 0.05; ∗∗*p* < 0.01; ∗∗∗*p* < 0.001; ns, not significant. Statistical significance was assessed using Welch’s *t* test. CS, cholesterol sulfate; SIS, sitosterol sulfate; CAS, campesterol sulfate; STS, stigmasterol sulfate; CNS, coprostanol sulfate.

### Bacterial Sults convert sitosterol to SIS

Previous reports have shown that the Sult from *Bacteroides thetaiotaomicron* produces CS, SIS, and CNS.^17^^,^^18^ SIS showed strong microbiota-dependence among phytosterol sulfates (Figures 1B and S2B), therefore, we investigated *in vitro* SIS production in 10 representative Bacteroidota species (Table 1). *P. goldsteinii*, *P. johnsonii*, *B. uniformis*, *B. fragilis*, *B. caccae*, and *B. thetaiotaomicron* produced both CS and SIS, whereas *A. finegoldii*, *P. gingivalis*, *B. intestinihominis*, and *P. denticola* did not produce either metabolite (Figure 2A). The CS-producing bacteria were limited to the *Bacteroides* and *Parabacteroides* genera within the Bacteroidota phylum. The observed peak of SIS was validated by spiking a SIS standard into lipid extracts from *B. thetaiotaomicron* (Figure 2B). Next, we investigated the presence of the *sult* gene in the cultured bacteria. Only two *sult* homologs of bacterial CS synthase from *B. thetaiotaomicron* and *Parabacteroides merdae* were reported.^17^^,^^18^ Homology searches using the amino acid sequence of *P. merdae sult* (*pmsult*) as a query revealed that six bacteria producing CS and SIS harbored a homologous *sult* sequence (*P. goldsteinii sult*: *pgsult*, *P. johnsonii*: *pjsult*, *B. thetaiotaomicron sult*: *btsult*, *B. caccae sult*: *bcsult*, *B. fragilis sult*: *bfsult*, *B. uniformis sult*: *busult*; Tables 1 and 2). The activities of the identified *sult* homologs were tested by heterologous expression in *Escherichia coli*. Protein expression was confirmed using western blotting (Figure 2C). Metabolic assays using *E. coli* lysates revealed that the expression of each Sult homolog resulted in SIS production (Figure 2D). These results demonstrate that bacterial SIS production by Sults is common in specific Bacteroidota species.

**Table 1:** Bacterial strains

Species	Identifier
*Bacteroides caccae*	JCM9498
*Bacteroides fragilis*	JCM11019
*Bacteroides uniformis*	JCM5828
*Bacteroides thetaiotaomicron*	JCM5827
*Parabacteroides goldsteinii*	JCM13446
*Parabacteroides johnsonii*	JCM13406
*Alistipes finegoldii*	JCM16770
*Prevotella denticola*	JCM13449
*Barnesiella intestinihominis*	JCM15079
*Porphyromonas gingivalis*	JCM12257

**Figure 2:**
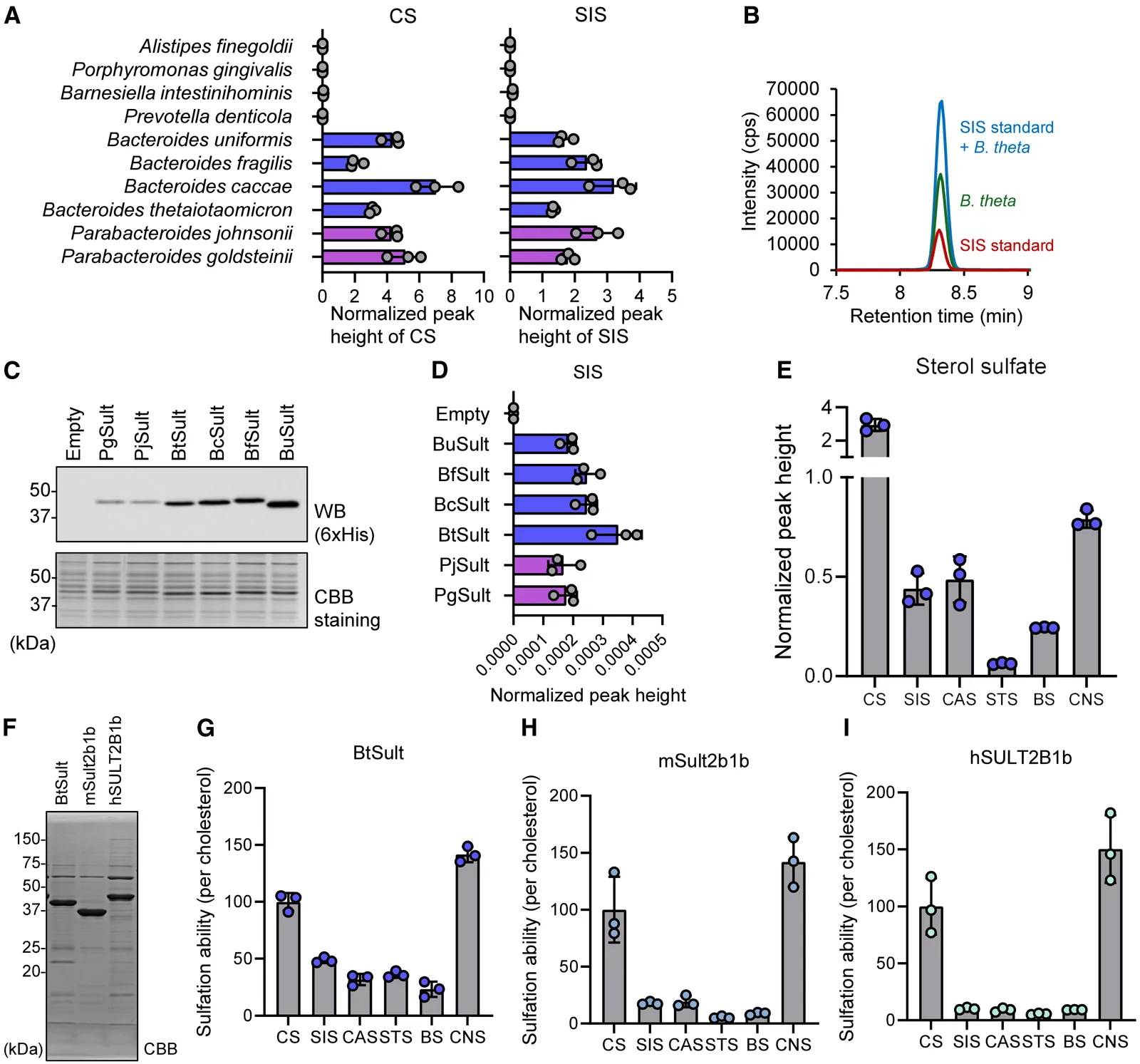
Characterization of SIS-producing bacteria and Sults (A) The normalized peak heights of CS and SIS. The 10 species of bacteria were anaerobically cultured with 10 μM cholesterol or sitosterol. Bars represent the mean, and error bars indicate SD. *n* = 3 independent bacterial cultures per condition. (B) The extracted ion chromatography of SIS. The chromatogram was extracted in a range of *m*/*z* = 493.34 ± 0.03 from each sample. (C) Western blot showing expression of the indicated Sult proteins in *E. coli* lysates. Image obtained from a single experiment. (D) Normalized peak heights of SIS. *E. coli* lysate protein (100 ng) was incubated with 50 μM sitosterol standard and 100 μM 3′-phosphoadenosine-5′-phosphosulfate (PAPS). Intensities were normalized by deviation from the internal standard and the Sult band intensity in CBB staining data. Bars represent the mean, and error bars indicate SD. *n* = 3 independent reactions. (E) Normalized peak heights of sterol sulfates. *Bacteroides thetaiotaomicron* was anaerobically cultured with 10 μM of each of six sterols. Bars represent the mean, and error bars indicate SD. *n* = 3 independent bacterial cultures per condition. (F) CBB-stained gel showing purified recombinant sulfotransferases. Image obtained from a single experiment. (G–I) Normalized peak heights of sterol sulfates. Purified sulfotransferase (1 μg) of (G) *B. thetaiotaomicron*, (H) mouse, and (I) human were incubated with 50 μM of each sterol standard and 100 μM of PAPS standard. Normalization was performed by dividing the raw intensities of sterol sulfates by the raw intensity of d7-lysophosphatidylcholine added as an internal standard. Bars represent the mean, and error bars indicate SD. *n* = 3 independent enzymatic reactions per condition. CS, cholesterol sulfate; SIS, sitosterol sulfate; CAS, campesterol sulfate; STS, stigmasterol sulfate; BS, brassicasterol sulfate; CNS, coprostanol sulfate; WB, western blotting; CBB, Coomassie brilliant blue.

**Table 2:** BLASTp results for searching *sult* homologs

	Species	Locus tag	Protein ID	Identities	Query cover
Query	*Parabacteroides merdae*	PARMER_01922	EDN86558.1	–	–
Subject	*Bacteroides caccae*	NQ555_10280	UNW77100.1	77%	100%
Subject	*Bacteroides fragilis*	E0L14_12615	QCT78192.1	75%	100%
Subject	*Bacteroides uniformis*	BACUNI_01338	EDO54816.1	76%	100%
Subject	*Bacteroides thetaiotaomicron*	BT_0416	AAO75523.1	76%	100%
Subject	*Parabacteroides goldsteinii*	HMPref. 1535_03910	KKB48682.1	95%	100%
Subject	*Parabacteroides johnsonii*	PRABACTJOHN_01528	EEC97068.1	98%	100%
Subject	*Alistipes finegoldii*	ND	ND	ND	ND
Subject	*Prevotella denticola*	ND	ND	ND	ND
Subject	*Barnesiella intestinihominis*	ND	ND	ND	ND
Subject	*Porphyromonas gingivalis*	ND	ND	ND	ND

ND, not detected.

We next evaluated whether *B. thetaiotaomicron* could produce other sterol sulfates. Culture experiments of *B. thetaiotaomicron* supplemented with each phytosterol confirmed the production of all the sterol sulfates tested (Figure 2E). Substrate-dependent differences in production efficiency were observed, with higher activity toward cholesterol and coprostanol, followed by campesterol, sitosterol, brassicasterol, and stigmasterol (Figure 2E). These findings suggest that other members of the Bacteroidota phylum harboring *sult* homologs may also contribute to sterol sulfate production.

The strong microbiota dependence of SIS raised the possibility that bacterial and mammalian sulfotransferases differ in their substrate preferences. To test this, we compared the substrate specificity of bacterial and mammalian sulfotransferases. Cholesterol, sitosterol, campesterol, stigmasterol, brassicasterol, and coprostanol were mixed with the recombinant BtSult, mouse Sult2b1b (mSult2b1b), and human SULT2B1b (hSULT2B1b), and the sulfation reaction by each enzyme was evaluated (Figure 2F). The BtSult, mSult2b1b, and hSULT2B1b converted cholesterol to CS as previously described.^17^^,^^23^^,^^24^ Notably, they also catalyzed phytosterol sulfation, including that of sitosterol, although their relative substrate preferences were different. When the CS quantity was normalized to 100%, the SIS production ratios were 51.6% for BtSult, 19.5% for mSult2b1b, and 11.4% for hSULT2B1b. BtSult also more efficiently converted other phytosterols to phytosterol sulfates than mSult2b1b and hSULT2B1b (Figures 2G–2I). Therefore, BtSult exhibited wide substrate specificity and can efficiently produce phytosterol sulfates when phytosterol substrates are provided in the diet.

### Microbiota-dependent tissue distribution of phytosterol sulfates

Bacteria-derived alkyne-tagged CS translocates into the hepatic portal vein in alkyne-tagged cholesterol fed mice.^18^ We hypothesized that, in contrast to unconjugated sitosterol, SIS is distributed to host tissues. To reveal the *in vivo* distribution of sterol sulfates, we reanalyzed a large-scale untargeted lipidomic dataset of multiple mouse tissues.^21^ This dataset included feces, plasma, and multiple organs collected from SPF and GF mice. The kidney, liver, spleen, lung, mesentery, small intestine, large intestine, skeletal muscle (tibialis anterior), back skin, and ear skin were analyzed. CS was detected across all analyzed tissues of 19-month-old male mice with no apparent dependence on the microbiota; rather, it tended to increase under GF conditions (Figures 3A and S3). Unconjugated cholesterol was also detected in all tissues; however, no clear trend was observed between SPF and GF conditions (Figures 3B and S6).

**Figure 3:**
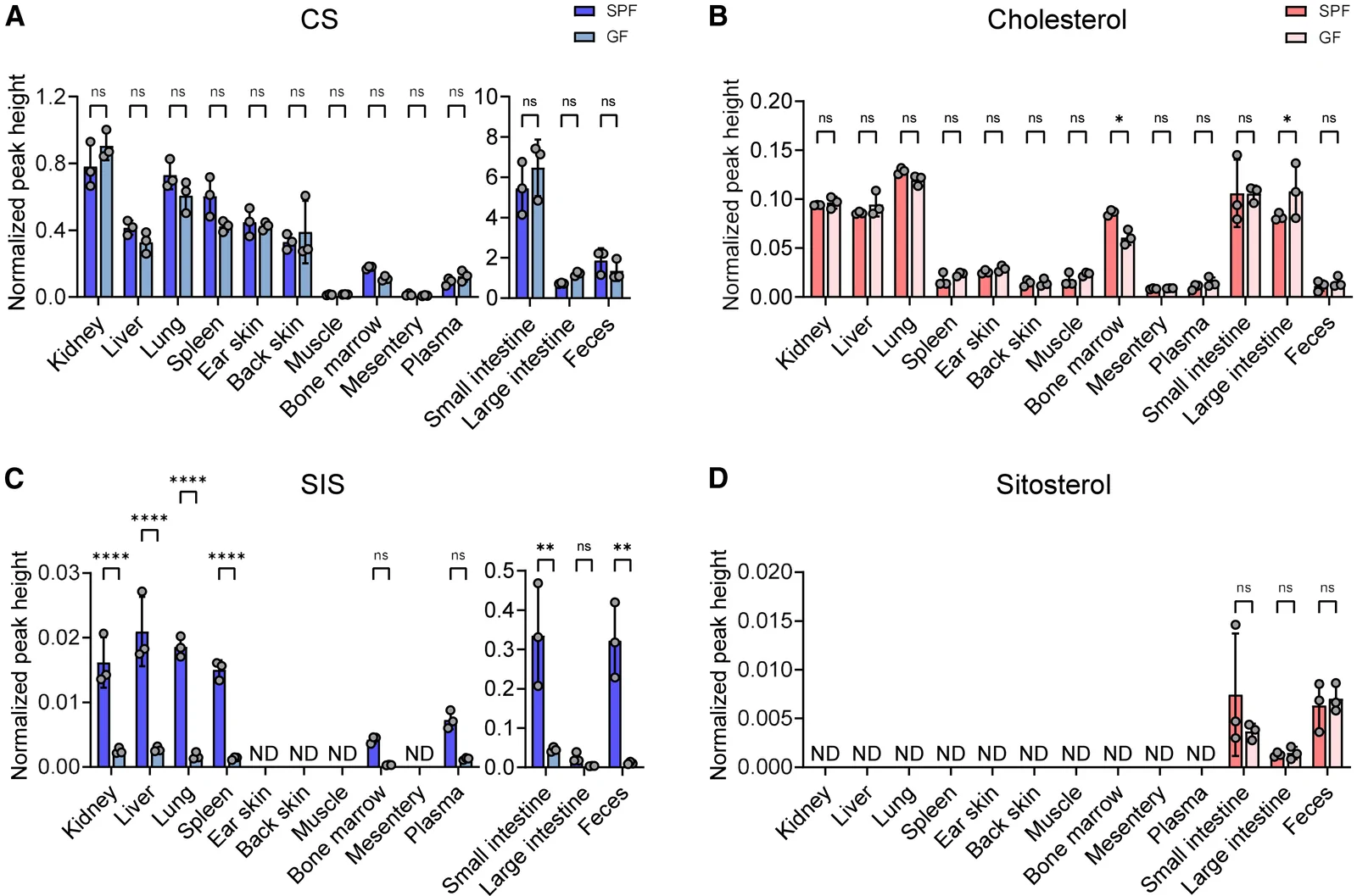
The microbiota dependency of sterol sulfates and sterols in 19-month-old male mouse tissues The normalized intensities of (A) CS, (B) cholesterol, (C) SIS, and (D) sitosterol. Normalization was performed by dividing the raw intensities of sterol sulfates by the raw intensity of d7-lysophosphatidylcholine and those of sterols by the raw intensity of d7-monoacylglycerol, both added as internal standards. Bars represent the mean, and error bars indicate SD. Each data point represents an individual mouse (*n* = 3 SPF mice and *n* = 3 GF mice). ∗*p* < 0.05; ∗∗*p* < 0.01; ∗∗∗∗*p* < 0.0001; ns, not significant; ND, not detected. Statistical significance was assessed using two-way ANOVA with microbiota status (SPF vs. GF) and tissue as factors, followed by Tukey’s multiple-comparisons test. CS, cholesterol sulfate; SIS, sitosterol sulfate; SPF, specific pathogen-free; GF, germ-free.

Notably, SIS was detected in nine tissues, including the liver, kidney, and plasma (Figures 3C and S3). In all tissues, the intensity of SIS was approximately 10% that of CS. Although CS was present in the skin, mesentery, and skeletal muscle, SIS was not detected in these tissues, highlighting the distinct tissue distributions of CS and SIS. Furthermore, SIS levels increased by more than 2-fold under SPF conditions. STS and CAS also exhibited similar tissue distribution patterns and microbiota dependence to those of SIS (Figure S4). Importantly, unconjugated sitosterol, stigmasterol, and campesterol levels were largely below the detection limit in these tissues (Figures 3D and S6), which is consistent with the finding that dietary sitosterol absorbed from the intestine is efficiently excreted via ABCG5/8.^25^ We also examined the levels of phytosterol sulfates in the AIN-93M diet fed to the SPF and GF mice in this dataset. However, no sterol sulfate peaks were detected (data not shown). Therefore, these data indicate that phytosterol sulfates accumulated in organs are not derived from the feed, but from the microbiota. BS was scarcely detected in extraintestinal tissues, possibly because of its low abundance (Figure S5). Therefore, phytosterol sulfates can translocate beyond the intestine and accumulate in host tissues in a gut microbiota-dependent manner.

### Age-dependent alterations of phytosterol sulfates

Next, we examined age-dependent changes in CS and phytosterol sulfate levels using the same dataset that was previously used to identify age- and microbiota-dependent lipid accumulation in host tissues.^21^ In the multi-organ dataset, CS levels did not exhibit significant age-dependent alterations in organs, such as the liver and kidney (Figure 4A). In contrast, SIS levels exhibited a 2- to 4-fold age-dependent increase in the bone marrow, kidney, liver, lung, plasma, and spleen, with a similar increasing trend observed in the small intestine, whereas no age-dependent increase was observed in CS levels (Figures 4A and 4B). No age-dependent changes were observed in cholesterol and sitosterol levels (Figures 4C, 4D, and S6). CAS and STS, which display gut microbiota dependence, also showed age-dependent increases in several organs, including the lungs and liver (Figure S4). Together, these results suggest that SIS, STS, and CAS are primarily synthesized by the gut microbiota within the intestinal lumen and subsequently absorbed into the host circulation, leading to systemic distribution in an age-dependent manner.

**Figure 4:**
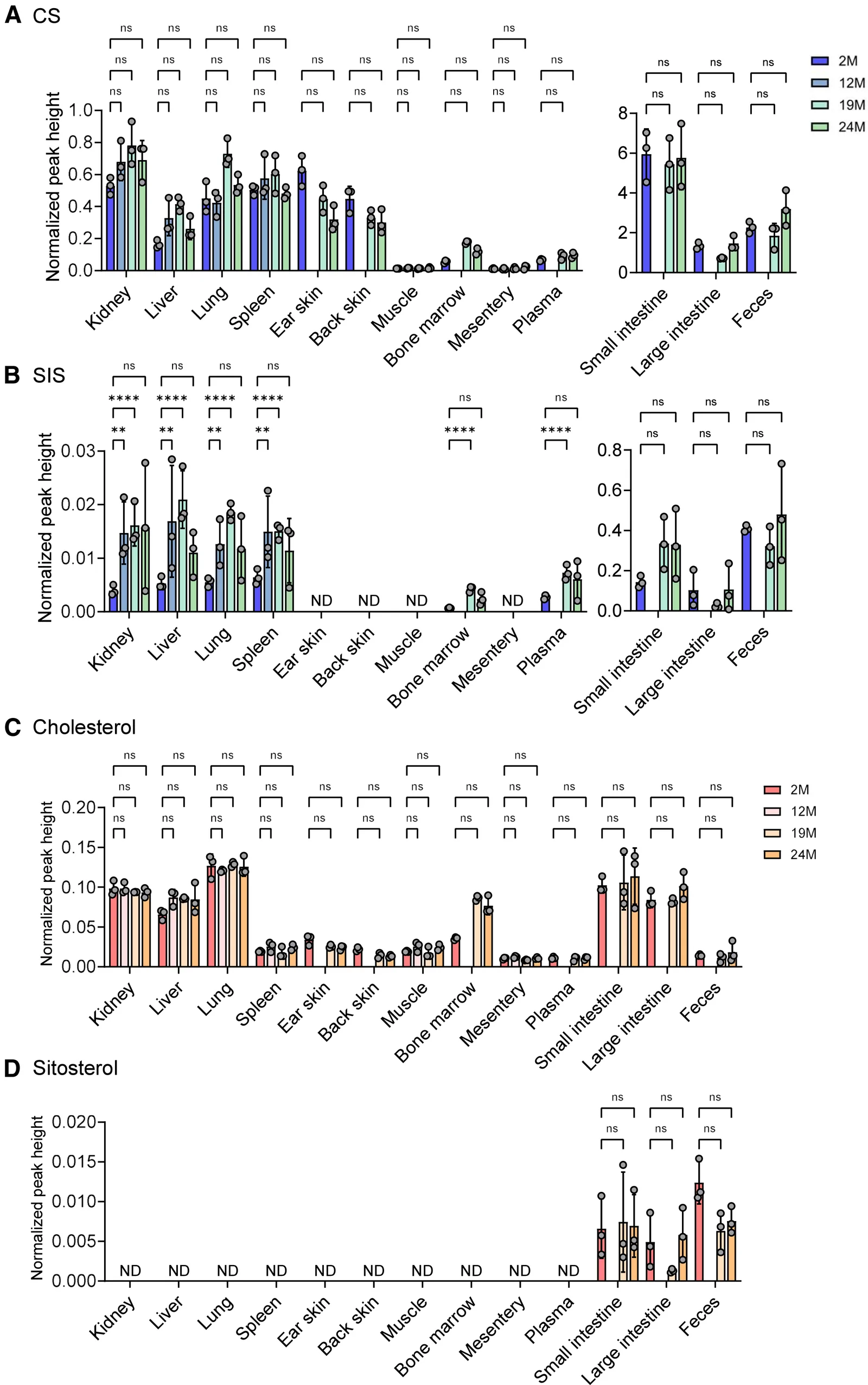
The age dependency of sterol sulfates and sterols in SPF mouse tissues The normalized intensities of (A) CS, (B) SIS, (C) cholesterol, and (D) sitosterol. The data of plasma, ear skin, back skin, bone marrow, small intestine, large intestine, and feces in 12-month-old mice were missing in datasets. Normalization was performed by dividing the raw intensities of sterol sulfates by the raw intensity of d7-lysophosphatidylcholine and those of sterols by the raw intensity of d7-monoacylglycerol, both added as internal standards. Bars represent the mean, and error bars indicate SD. Each data point represents an individual mouse; the number of mice analyzed for each age and tissue is indicated in the figure (*n* = 3 per group). ∗∗*p* < 0.01; ∗∗∗∗*p* < 0.0001; ns, not significant; ND, not detected. Statistical significance was assessed using two-way ANOVA with age of mice and tissue as factors, followed by Tukey’s multiple-comparisons test. CS, cholesterol sulfate; SIS, sitosterol sulfate; SPF, specific pathogen-free.

SIS was detected in human serum lipidomics,^26^ therefore, we reanalyzed human cohort lipidomics data. In addition to CS, signals for SIS, CAS, and STS were observed in human plasma (Figures S7A and S7C–S7F). BS and CNS were also detected in the fecal samples (Figure S7B). Therefore, phytosterol sulfates systemically circulate in humans, similar to those in mice.

Collectively, these findings show that phytosterol sulfates are systemically distributed in mice in a microbiota-dependent manner. SIS, CAS, and STS detection in the human plasma further supports the translational relevance of these observations.

## Discussion

In this study, we demonstrated that phytosterol, which is generally considered poorly absorbed and efficiently excreted by the host, can be converted into its sulfated form in a gut microbiota-dependent manner. Among the phytosterol sulfates detected, SIS was detected at the highest abundance in host tissues in an age-dependent manner. While non-sulfated sitosterol was largely undetectable in peripheral tissues, SIS showed a broad tissue distribution that was markedly reduced in GF mice, indicating a substantial contribution of the gut microbiota in shaping the host sterol sulfate pool. These findings challenge the prevailing view that phytosterols are biologically inert dietary components, revealing a previously unrecognized route by which gut microbes transform dietary sterols into metabolites that persist within the host.

Previous studies have identified seven bacterial species capable of producing CS, including *B*. *thetaiotaomicron*, *B*. *fragilis*, *B*. *ovatus*, *B*. *caccae*, *B*. *uniformis*, *B*. *vulgatus*, and *P*. *merdae*.^17^^,^^18^ In the present study, we confirmed CS and SIS production by four previously reported CS-producing species (*B. thetaiotaomicron*, *B. fragilis*, *B. caccae*, and *B. uniformis*). The identification of additional sterol sulfate-producing species also provides a broader framework for investigating how inter-individual differences in gut microbial composition may influence phytosterol metabolism and the systemic exposure to microbiota-derived sterol sulfates. The physiological importance of bacterial *sult* genes remains unclear. The *sult* knockout strain showed no reduction in colonization fitness in GF mice compared to the wild-type strain, exhibited no difference *in vitro* growth rates, and displayed no alteration in viability across a range of exogenous cholesterol concentrations.^18^ These observations collectively suggest that cholesterol does not exert overt toxicity on these bacteria and that sterol sulfation is not required for bacterial survival under the conditions tested. The precise physiological role of sterol sulfation in bacteria thus remains an open and important question that warrants further investigation.

A previous study showed that alkyne-labeled bacterial CS is present in the hepatic portal vein, demonstrating that microbially generated sterol sulfates can translocate beyond the intestine.^18^ Therefore, the distribution observed in tissues may occur via the hepatic portal vein circulation. Furthermore, this study revealed that colonizing GF mice with the *B*. *thetaiotaomicron* BT0416 (*btsult*) knockout strain resulted in reduced plasma cholesterol concentrations compared to colonization with the wild-type strain. Therefore, sterol sulfates derived from the gut bacteria may influence cholesterol homeostasis and the molecular entities involved are diverse.

Recent work has established that gut bacteria possess sulfotransferases capable of catalyzing sterol sulfation, and these microbial activities can influence the fecal and circulating levels of CS.^17^ However, previous studies have largely focused on host-derived sterols and have not addressed whether microbial sulfation of phytosterols alters host disposition. Our data extend these emerging insights by showing that microbial sterol sulfation applies to phytosterols, with clear consequences for their tissue distribution. Six Bacteroidota species were identified as the SIS-producing bacteria. These six species are major members of the human gut microbiota.^27^^,^^28^ The presence of SIS in human feces and plasma suggests that systemic distribution of SIS also occurs in humans. Serum levels of SIS are increased in patients with Crohn’s disease (CD), indicating its potential as a biomarker.^26^ Since dysbiosis occurs in patients with CD, the elevation in SIS may reflect changes in the gut microbiota.^29^^,^^30^ Compared to BtSult, mSult2b1b and hSULT2B1b exhibit much lower efficiency to produce phytosterol sulfates, and based on the abundance of phytosterol substrate provided from the diet, it is suggested that phytosterol sulfates present in host tissues are mostly derived from gut microbiota. Thus, the marked elevation in serum SIS levels in patients with CD may be associated with characteristic gut dysbiosis.

This study confirmed *in vitro* that sterol-supplemented BtSult produced all sterol sulfates detected in mouse feces and tissues. However, there is a clear difference in substrate specificity between bacterial and mammalian sulfotransferase enzymes. Biochemical validation and docking simulations using the hSULT2B1b crystal structure suggested that the C-17 alkyl group of cholesterol directly contacts the Ile20 residue of hSULT2B1b, thereby contributing to substrate specificity.^31^^,^^32^ Although there are no verified examples of phytosterol recognition by hSULT2B1b, bacterial Sults are likely to have more space in the catalytic site to accommodate the bulky side chains of sterol substrates, including phytosterols.

Intestinal cholesterol homeostasis is tightly regulated by coordinated uptake, trafficking, esterification, and efflux pathways. Dietary cholesterol and phytosterols are taken up via Niemann-Pick C1-like 1 (NPC1L1),^9^^,^^11^ trafficked intracellularly by Niemann-Pick C1 (NPC1) and NPC2,^33^ esterified by acyl-CoA-cholesterol acyltransferase1/2 (ACAT1/2) for chylomicron assembly,^16^^,^^25^ or expelled back into the lumen by ATP-binding cassette transporters (ABCG5/ABCG8).^12^^,^^13^ Under normal conditions, sitosterol is not incorporated into chylomicrons because it is poorly esterified by ACAT2 and is efficiently excluded from the systemic circulation via ABCG5/ABCG8-mediated efflux.^34^

In contrast, SIS accumulation in host tissues suggests that sulfation fundamentally alters sterol trafficking and disposition. One plausible mechanism involves the NPC pathway, as CS interacts with NPC proteins and modulates sterol transport.^35^^,^^36^ Similarly, SIS may be recognized by NPC2 or related sterol-binding proteins, promoting intracellular retention or altered redistribution. At the absorption level, it remains unclear whether NPC1L1 directly transports sulfated sterols. However, the presence of SIS in the peripheral tissues indicates that sulfation may redirect sterols into alternative uptake or transport routes. Moreover, because ABCG5/ABCG8 preferentially transports neutral sterols, sulfation-induced polarity changes may allow SIS to evade canonical exclusion mechanisms, facilitating its persistence in host tissues.

The mechanisms underlying the age-dependent accumulation of SIS via absorption, systemic circulation, degradation, and excretion from the intestinal tract remain unclear. However, our data showed that SIS in the feed was below the detection limits and producing bacteria did not increase in an age-dependent manner (data not shown). Contributing factors may include age-dependent intestinal barrier disruption, systemic decline in digestive enzyme function, or reduced renal or biliary excretion. Steroid sulfatase gene deficiency accelerated cell senescence *in vitro*,^37^ suggesting that steroid sulfate accumulation contributes to senescence in mice.

The physiological relevance of SIS accumulation in the body is unclear. However, based on its structural similarity to CS, SIS may interact with CS-interacting proteins, such as macrophage-inducible C-type lectin (Mincle) and dedicator of cytokinesis protein 2 (DOCK2) as alternative ligands.^5^^,^^6^ Notably, in sitosterolemia, excessive accumulation of non-sulfated phytosterols leads to diverse clinical manifestations, including premature atherosclerosis.^13^ Our findings suggest that SIS accumulation may have detrimental effects on the host. This hypothesis is based on the possibility that SIS may activate the immune system, similar to CS, and that free sitosterol may be generated in target tissues.

Collectively, our findings have implications for sterol metabolism and homeostasis in humans. Untargeted lipidomic studies detected CS, SIS, and related sterol sulfates in human plasma, indicating that these metabolites systemically circulate in humans.^26^ By demonstrating that representative human gut bacteria can produce a broad spectrum of sterol sulfates, our study provides experimental evidence of the microbial origin of these circulating molecules and establishes a foundation for future mechanistic studies. Elucidating the transport, degradation, and molecular targets of SIS is essential to understand how dietary phytosterols, gut microbiota, and host metabolism relate to physiology and disease.

### Limitations of the study

This study has several limitations. First, only a limited number of bacterial strains were examined. Second, only male mice were included in the present analysis. Therefore, potential sex-dependent differences remain unknown. Third, the ADME (absorption, distribution, metabolism, and excretion) pathway of sterol sulfates is unclear. Finally, although our results demonstrate that gut bacteria can generate sulfated plant sterols, the physiological effects of these bacteria-derived sterol sulfates on the host remain unknown. Further studies will be required to clarify the physiological relevance of this pathway *in vivo* using a gnotobiotic system colonized with *sult*-knockout or wild-type bacterial strains and fed phytosterols.

## Resource availability

### Lead contact

Requests for further information and resources or reagents should be directed to and will be fulfilled by the lead contact, Makoto Arita (marita@keio.jp).

### Materials availability

Plasmids generated in this study are available upon request.

### Data and code availability

All data in this paper are available in MB-POST. The mass spectrometry data have been deposited in the MB-POST: MPST000142, MPST000143, MPST000144, MPST000145, and MPST000160. All original code is available from the lead contact upon request. Any additional information required to reanalyze the data reported in this paper is available from the lead contact upon request.

## Acknowledgments

This work was supported by the JST-ERATO “ARITA Lipidome Atlas Project” under grant number JPMJER2101 (M.A.), AMED Moonshot Research and Development Program JP22zf0127007 (M.A.), AMED NeDDTrim (21ae0121036 to M.A.), and World Premier International Research Center Initiative - Human Biology-Microbiome-Quantum Research Center (WPI-Bio2Q). This work was also supported by the Keio University Doctorate Student Grant-in-Aid Program (K.T.) and the Keio JST-SPRING program (K.T.). We thank the Riken BioResource Center for providing bacterial strains, Twist Bioscience for synthesizing genes, and Editage (www.editage.jp) for English language editing. The graphical abstract was created with BioRender.com (https://BioRender.com/ap3aq4w).

## Author contributions

Conceptualization, M.A.; methodology, K.T. and S.M.; software, K.T. and S.M.; validation, K.T. and T.N.; formal analysis, K.T.; investigation, K.T. and T.N.; writing – original draft preparation, K.T.; writing – review and editing, S.M., N.O., and M.A.; visualization, K.T.; supervision, S.M. and M.A.; funding acquisition, M.A. All authors have read and agreed to the published version of the manuscript.

## Declaration of interests

The authors declare no competing interests.

## Declaration of generative AI and AI-assisted technologies in the manuscript preparation process

During the preparation of this work, the authors used ChatGPT with GPT-5 (OpenAI) only to assist in drafting Python code for visualization of MS/MS spectra. The underlying data were not altered by the use of ChatGPT. The generated output was reviewed by the authors, who take full responsibility for the content of the publication.

## STAR★Methods

### Key resources table

**Table:** 

REAGENT or RESOURCE	SOURCE	IDENTIFIER
**Antibodies**		

Anti 6×Histidine, Monoclonal Antibody(9C11)	Wako	Cat# 015-23094; RRID: AB_2728686
Mouse IgG HRP Linked F(ab’)2 Fragment	Cytiva	Cat# NA9310; RRID: AB_772193

**Bacterial and virus strains**		

*Bacteroides thetaiotaomicron*	RIKEN BRC	JCM5827
*Bacteroides caccae*	RIKEN BRC	JCM9498
*Bacteroides fragilis*	RIKEN BRC	JCM11019
*Bacteroides uniformis*	RIKEN BRC	JCM5828
*Prevotella denticola*	RIKEN BRC	JCM13449
*Alistipes finegoldii*	RIKEN BRC	JCM16770
*Parabacteroides goldsteinii*	RIKEN BRC	JCM13446
*Parabacteroides johnsonii*	RIKEN BRC	JCM13406
*Barnesiella intestinihominis*	RIKEN BRC	JCM15079
*Porphyromonas gingivalis*	RIKEN BRC	JCM12257
ECOS^TM^ Competent *Escherichia coli* JM109	Nippon Gene	Cat# 311-06244
ECOS^TM^ Competent *Escherichia coli* BL21 (DE3)	Nippon Gene	Cat# 312-06534

**Chemicals, peptides, and recombinant proteins**		

RIPA buffer	Nacalai Tesque	Cat# 16488-34
0.1 mol/L Phosphate Buffer Solution, pH6.8	Wako	Cat# 161-27145
PBS, pH 7.4	Gibco™	Cat# 10010023
cOmplete™, Mini, EDTA-free Protease Inhibitor Cocktail	Roche	Cat# 04693159001
Clarify Western ECL Substrate	Bio-Rad	Cat# 1705061
Acudia^TM^ Modified GAM Broth	Shimadzu Diagnostics	Cat# 05422
Acudia^TM^ Modified GAM Agar	Shimadzu Diagnostics	Cat# 05420
LB Broth Mirror (Luria-Bertani)	BD Difco^TM^	Cat# 244620
Recombinant mouse Sult2b1	This paper	N/A
Recombinant human SULT2B1	This paper	N/A
Recombinant *Bacteroides thetaiotaomicron* Sult	This paper	N/A
3′-Phosphoadenosine-5′-phosphosulfate triethylammonium salt	Sigma-Aldrich	Cat# 94455-1 MG
Methanol	Wako	Cat# 138-18541
EquiSPLASH™	Avanti Research	Cat# 330731-1 EA
Sitosterol sulfate (trimethylamine)	MedChemExpress	Cat# HY-N10478A
Cholesterol	Sigma-Aldrich	Cat# C8667-5G
β-Sitosterol, synthetic, ≥95%	Sigma-Aldrich	Cat# S1270-25 MG
Brassicasterol	TAMA BIOCHEMICAL	Cat# 306-02871
Campesterol	TAMA BIOCHEMICAL	Cat# 306-01391
Stigmasterol	TAMA BIOCHEMICAL	Cat# 302-01413
Coprostanol	Sigma-Aldrich	Cat# C7578-50 MG

**Critical commercial assays**		

In-Fusion® HD Cloning Kit	TaKaRa	Cat# 639649
Pierce™ BCA Protein Assay Kit	Thermo Fisher Scientific	Cat# 23225
Wizard SV Gel and PCR Clean-Up System	Promega	Cat# A9282
NucleoSpin® Plasmid EasyPure	TaKaRa	Cat# 740727.250
NucleoBond® Xtra Midi/Maxi	TaKaRa	Cat# 740410.100

**Deposited data**		

Untargeted lipidomics data	This study	MB-POST: MPST000142, MPST000143, MPST000144, MPST000145, MPST000160

**Oligonucleotides**		

Forward primer to amplify *sult* of *Bacteroides thetaiotaomicron* GAAGGAGATATACATA TGGGATTACTTGAATTTAATAAAC	Sigma-Aldrich	N/A
Reverse primer to amplify *sult* of *Bacteroides thetaiotaomicron* GTGGTGGTGCTCGAG TAAATTATAGTTCCATTGTTTCAAA	Sigma-Aldrich	N/A
Forward primer to amplify *sult* of *Bacteroides fragilis* GAAGGAGATATACATATGG GATTACTTGAATTTAACAAAC	Sigma-Aldrich	N/A
Reverse primer to amplify *sult* of *Bacteroides fragilis* GTGGTGGTGCTCGAGTAAAC TATAATCCCATTGTTTCAGT	Sigma-Aldrich	N/A
Forward primer to amplify *sult* of *Bacteroides uniformis* GAAGGAGATATACATATGGGA TTGTTAGAATTCAATAAGC	Sigma-Aldrich	N/A
Reverse primer to amplify *sult* of *Bacteroides uniformis* GTGGTGGTGCTCGAGTAA ATCGTACTTCCACTGTTCCAGC	Sigma-Aldrich	N/A
Forward primer to amplify *sult* of *Bacteroides caccae* GAAGGAGATATACATA TGGGATTACTCGAATTTAATAAAC	Sigma-Aldrich	N/A
Reverse primer to amplify *sult* of *Bacteroides caccae* GTGGTGGTGCTCGAGTAAAT TATAATCCCATTGTTTCAAA	Sigma-Aldrich	N/A
Forward primer to amplify *sult* of *Parabacteroides goldsteinii* GAAGGAGATATACATATGGGGT TGCTGGAATTTGATAAAT	Sigma-Aldrich	N/A
Reverse primer to amplify *sult* of *Parabacteroides goldsteinii* GTGGTGGTGCTCGAGTAAAGA ATATCCCCACTCCTTGAGA	Sigma-Aldrich	N/A
Forward primer to amplify *sult* of *Parabacteroides johnsonii* GAAGGAGATATACATATGGGAT TACTGGAATTTGACAAAC	Sigma-Aldrich	N/A
Reverse primer to amplify *sult* of *Parabacteroides johnsonii* GTGGTGGTGCTCGAGTAAAGA ATATCCCCACTCCTTGAGA	Sigma-Aldrich	N/A
Forward primer to amplify mouse *Sult2b1* GAAGGAGATATACATAT GGACGGCCCGCAGCCGCG	Sigma-Aldrich	N/A
Reverse primer to amplify mouse *Sult2b1* GTGGTGGTGCTCGAG CTGGGATGAACCCGGATTCGG	Sigma-Aldrich	N/A
Forward primer to amplify human *SULT2B1* GAAGGAGATATACATA TGGATGGCCCGGCGGAACCGCAAA	Sigma-Aldrich	N/A
Reverse primer to amplify human *SULT2B1* GTGGTGGTGCTCGAGC GATGGGCGCGGATG	Sigma-Aldrich	N/A

**Recombinant DNA**		

pET21a (+)	Addgene	Cat#69740-3
pET21a (+)-BtSult	This study	N/A
pET21a (+)-BfSult	This study	N/A
pET21a (+)-BuSult	This study	N/A
pET21a (+)-BcSult	This study	N/A
pET21a (+)-PgSult	This study	N/A
pET21a (+)-PjSult	This study	N/A
Codon optimized mouse Sult2b1	Twist bioscience	N/A
Codon optimized human SULT2B1	Twist bioscience	N/A
pET21a (+)-mouse Sult2b1	This study	N/A
pET21a (+) -human SULT2B1	This study	N/A

**Software and algorithms**		

ImageJ.JS version 0.6.0	ImageJ development team	version 0.6.0; https://imagej.net
GraphPad Prism version 10.3.1	GraphPad Software	version 10.3.1; https://www.graphpad.com
MS-DIAL version 5.1.231129-dev	RIKEN Center for Sustainable Resource Science	version 5.1.231129-dev; https://github.com/systemsomicslab/MS-DIAL
Python version 3.11	Python Software Foundation	version 3.11; https://www.python.org
Multiple Sequence Alignment (MSA)	Robert C. Edgar	https://www.drive5.com/muscle

### Experimental model and study participant details

#### Reanalysis of the published data

Untargeted lipidomic datasets of control and antibiotic-treated mouse feces (RIKEN DROPMet: DM0032),^22^ specific pathogen-free (SPF) and germ-free (GF) mice (RIKEN DROPMet: DM0044),^21^ and human feces and plasma (RIKEN DROPMet: DM0037)^38^ were obtained from RIKEN DROPMet (https://prime.psc.riken.jp/menta.cgi/prime/drop_index) and analyzed using MS-DIAL version 5.1.231129-dev.^39^

#### Mice

No new animal experiments were performed in this study. The mouse data analyzed in this study were obtained from previously published datasets deposited in RIKEN DROPMet. For the control and antibiotic-treated mouse dataset, the original study used male wild-type C57BL/6J mice obtained from CLEA Japan, Inc. (Tokyo, Japan) and maintained under specific pathogen-free (SPF) conditions. Antibiotic-treated mice received ampicillin (1 g/L), vancomycin (0.5 g/L), neomycin (1 g/L), and metronidazole (1 g/L) in the drinking water for two weeks from six weeks of age, while control mice received drinking water without antibiotics. Five mice were included in each group. For the SPF and germ-free (GF) mouse dataset, the original study used four-week-old wild-type C57BL/6N mice obtained from CLEA Japan, and GF mice were housed in GF isolators at the RIKEN animal facility. Only male mice were included in the present analysis. The original animal experiments were approved by the Animal Care and Use Committee of RIKEN Center for Integrative Medical Sciences (2019-015(2)).

#### Human study participants

This study involved the reanalysis of previously published human fecal and plasma lipidomics data obtained from RIKEN DROPMet. The original study recruited Japanese men and women aged 20–75 years during annual health check-ups at the University of Tokyo Hospital between 2014 and 2016. Among the enrolled participants, 112 were classified as healthy, 100 as obese, and 101 as having a prediabetic condition. Only the healthy participants were included in the present analysis, and lipidomics data were available for 107 of the 112 healthy participants (*n* = 107). The original study obtained written informed consent from the participants and was approved by the institutional review boards of RIKEN and The University of Tokyo.

#### Intestinal bacterial culture

Bacterial strains were obtained from RIKEN BRC (Ibaraki, Japan). An anaerobic chamber (Coy Laboratory Products, Grass Lake, MI, USA) was maintained under the following conditions: oxygen concentration less than 5 ppm; hydrogen concentration, 10%; carbon dioxide, 10%; nitrogen, 80%; and humidity, 60%. All bacteria except *Escherichia coli* were cultured under anaerobic conditions in modified Gifu Anaerobic Medium (mGAM, Shimadzu Diagnostics, Tokyo, Japan). The glycerol stock was inoculated on mGAM agar and incubated for 1–3 days until colonies formed. Single colonies were inoculated in 1 mL of mGAM broth and incubated for 1–2 days at 37°C. From that culture, 250 μL was inoculated into 10 mL of mGAM broth containing 10 μM standards and incubated for 18–20 h at 37°C.

### Method details

#### *Sult* cloning and heterologous expression in *E. coli*

The amino acid and nucleotide sequences of *Parabacteroides merdae* (PARMER_01922; UniProt accession number: X83954) were obtained from the NCBI database. The *pmsult* homologous gene sequences were searched using local BLASTp with BLAST+ version 2.16.0 constructed in the Linux environment. Sequences that satisfied query cover >80%, e-value < 1E−5, and amino acid length >300 were considered *sult* homologs (Table 2). Bacteroidota-derived *sult* sequences were amplified using PCR with the bacterial strains as templates and primer pairs (Table S1). The mouse *sult2b1* and human *SULT2B1* genes were codon-optimized for the *E. coli* B strain and synthesized using Twist Bioscience (San Francisco, CA, USA). The adaptor DNA sequences for the In-Fusion reaction were attached to the synthetic genes using the primers. The PCR products were cloned into pET-21a(+) (Addgene, Watertown, MA, USA) expression vector using In-Fusion reaction (In-Fusion® HD Cloning Kit, Takara Bio Inc., Shiga, Japan) with a C-terminal 6×-histidine tag.^40^ Plasmids were introduced into ECOS^TM^ Competent *E. coli* BL21 (DE3) cells (Nippon Gene, Tokyo, Japan). Transformants were inoculated onto Luria–Bertani (LB) agar plates (BD Difco^TM^, Franklin Lakes, NJ, USA) containing 100 μg/mL ampicillin (Fujifilm Wako Pure Chemical Corporation, Osaka, Japan). Single colonies were inoculated in 1 mL of LB broth containing ampicillin and incubated overnight at 37°C at 220 rpm shaking as a pre-culture.

#### *In vitro* assay of *E. coli* lysate expressing Bacteroidota-derived Sult

A 100 μL pre-culture broth was inoculated into 3 mL of LB broth. The mixture was then incubated for 18 h at 37°C at 220 rpm shaking. The cells were then collected using centrifugation at 5,000 × g for 30 min at 4°C. The *E. coli* pellet of the 3-mL culture was suspended in 200 μL of 50 mM phosphate buffer, pH 7.2, and 150 mM NaCl (phosphate buffer) supplemented with cOmplete™ Protease Inhibitor Cocktail (Roche Diagnostics Deutschland GmbH, Mannheim, Germany). The samples were sonicated 15 times using a probe sonicator Q125 (QSONICA, Newtown, CT, USA) at 20% amplitude. The supernatant was then centrifuged at 18,000 × g for 1 min at 4°C to remove the debris. The supernatant was quantified using the Pierce^TM^ BCA Protein Assay Kit (Thermo Fisher Scientific, Waltham, MA, USA). Coomassie Brilliant Blue (CBB) -stained protein bands were quantified using the Gel Analyzer tool in ImageJ.JS (version 0.6.0). Each lane was selected using a region of interest (ROI), and the peak area corresponding to each protein band in the lane profile was used as the band intensity. Background subtraction was not performed. Protein concentrations were estimated using a calibration curve generated from BSA standards at 0.0625, 0.125, 0.25, 0.5, 1, and 2 μg. Protein (100 ng) was added to 100 μL of phosphate buffer containing 100 μM 3′-phosphoadenosine-5′-phosphosulfate (PAPS) and 50 μM sitosterol and incubated for 4 h at 37°C.

#### *In vitro* assay of purified polyhistidine-tagged sulfotransferases

A 1 mL pre-culture broth was inoculated into 1 L of LB broth. The mixture was then incubated for 18 h at 37°C at 220 rpm shaking for *E. coli* containing pET21a(+)-*btsult*. The mixture was then incubated at an OD_600_ of approximately 0.2, to which 0.1 mM IPTG was added, and shaken at 220 rpm for 4 h at 37°C for *E. coli* containing pET21a(+)-*sult2b1* and pET21a(+)-*SULT2B1*. The cells were then collected using centrifugation at 5,000 × g for 30 min at 4°C. The *E. coli* pellet of the 1 L culture was suspended in 10 mL of phosphate buffer supplemented with cOmplete™ Protease Inhibitor Cocktail (Roche Diagnostics Deutschland GmbH). The samples were sonicated using a probe sonicator (Q125, QSONICA) at 40% amplitude for 60 cycles of 10 s each, with 50 s intervals between pulses. Then, the samples were centrifuged at 18,000 × g for 10 min at 4°C and the supernatants were transferred to new tubes and centrifuged again under the same conditions to remove the debris. The supernatants were incubated with Ni Sepharose™ High Performance (Cytiva, Marlborough, MA, USA) for 1 h at 4°C. The resin was washed with phosphate buffer containing 50 mM imidazole and proteins were eluted using phosphate buffer containing 500 mM imidazole. The eluate was concentrated using an Amicon Ultra centrifugal filter (30 kDa molecular weight cut-off, Merck Millipore) to approximately 500 μL. Quantification was performed using a Pierce^TM^ BCA Protein Assay Kit (Thermo Fisher Scientific). The purity of sulfotransferases was quantified using a calibration curve generated from CBB-stained BSA standards at 0.0625, 0.125, 0.25, 0.5, 1, and 2 μg, as described above. BtSult was selected as the representative bacterial sulfotransferase for comparative analysis because it is a well-characterized CS-producing enzyme and the only bacterial Sult homolog for which His-tag-based isolation and purification has been previously demonstrated, making it the most technically feasible candidate for direct comparison with the mammalian enzymes. The purity of the recombinant proteins used in this paper was comparable to that reported in previous studies.^17^^,^^18^ Sulfotransferase (1 μg) was added to 100 μL of phosphate buffer containing 50 μM of each sterol standard and 100 μM of PAPS, and incubated for 24 h at 37°C.

#### CBB staining and western blotting

The quantified protein lysate was mixed with sodium dodecyl sulfate buffer and incubated for 10 min at 25°C. After sodium dodecyl sulfate polyacrylamide gel electrophoresis using 12.5% gels, the gels were stained with CBB Page Blue 83 Stain Reagent (CBB-R250; Cosmo Bio, Tokyo, Japan). Detection was performed using a ChemiDoc^TM^ Touch Imaging System (Bio-Rad, Hercules, CA, USA). For western blotting, the proteins were transferred from the gels to nitrocellulose membranes (Bio-Rad) using a Trans-Blot Turbo (Bio-Rad). After blocking with 5% skim milk in Tris-buffered saline containing 0.05% Tween 20, the washed membrane was immersed in an anti-6× Histidine, Monoclonal Antibody (9C11) (Fujifilm Wako Pure Chemical Corporation). The membranes were then immersed in horseradish peroxidase-labeled anti-mouse IgG antibody (Cytiva). Detection was performed using Clarity Western ECL Substrate (Bio-Rad) and a ChemiDoc^TM^ Touch Imaging System (Bio-Rad).

#### Lipid extraction

For bacterial lipidomics, 195 μL of methanol, 5 μL of EquiSPLASH (Avanti Research) and 100 μL of chloroform were added per 1 mg dried bacterial pellet, followed by incubation for 2 h at RT. Then, 20 μL of water per 1 mg pellet was added and centrifuged at 2,000 × g for 10 min^41^ The supernatant was transferred to a measurement vial and analyzed using liquid chromatography-mass spectrometry.

For lipidomics of reaction products of Sulfotransferase-expressed *E. coli* lysates and purified sulfotransferases, a 100 μL reaction solution in phosphate buffer was mixed with 245 μL of methanol, 5 μL of EquiSPLASH (Avanti Research) and 125 μL of chloroform, followed by incubation for 10 min. Then, 125 μL of water and 125 μL of chloroform were added, vortex mixed, and centrifuged at 2,000 × g for 10 min. The upper phase was removed and lower layer was dried using a centrifugal concentrator (TAITEC, Saitama, Japan). The dried lipids were dissolved in a 320 μL solution (methanol:chloroform:water = 200:100:20 *v/v/v*).

For lipidomics of mouse food, 100 μL of methanol were added per 50 mg food, and homogenized at 2000 rpm for 15 s. The supernatant was transferred to a new vial and frozen at −80°C overnight. Then, 40 μL of sample solvent was transferred to a new glass tube and 155 μL of methanol, 5 μL of EquiSPLASH (Avanti Research) and 100 μL of chloroform were added. The glass tube was vortexed for 10 s and incubated at 25°C for 2h. After incubation, 20 μL of water was added and incubated at 25°C for 10 min. The glass tube was centrifuged at 2,000 × g for 10 min. The supernatant was transferred to an LC vial.

#### Lipidomics

Bacterial lipidomics was performed using an LC system (Nexera; Shimadzu, Kyoto, Japan) coupled to a quadrupole/time-of-flight MS (ZenoTOF 7600; SCIEX, Framingham, MA, USA).^12^^,^^29^ Lipids were separated using a YMC-Triart C18 column (50 × 2.1 mm,1.9 μm, 12 nm; YMC, Kyoto, Japan) using mobile phase (A) 1:1:3 (*v/v/v*) acetonitrile:methanol:water, and (B) 9:1 (*v/v*) 2-propanol:acetonitrile. Both the solvents were supplemented with 5 mM ammonium acetate and 10 nM ethylenediaminetetraacetic acid. The column was maintained at 45°C and a flow rate of 0.3 mL/min. A sample volume of 1 μL was used for injection. The gradient method was as follows: 0 min 0% (B); 1 min 0.5% (B); 4 min 40% (B); 7.5 min 64% (B); 12 min 71% (B); 12.5 min 82.5% (B); 19 min 85% (B); 20 min 99% (B); 22 min 99% (B); 22.1 min 0.5% (B); and 25 min 0.5% (B). The normalized sterol sulfates intensities were calculated using deuterium-labeled lysophosphatidylcholine (18:1-d7-LPC; Avanti Research, Alabaster, AL, USA) as an internal standard. The normalized sterols intensities were calculated using deuterium-labeled monoacylglycerol (18:1-d7-MG; Avanti Research) as an internal standard.

Mouse food lipidomics was performed following the experimental procedures described in a previous paper.^21^ Lipidomics was performed using a Waters ACQUITY ultraperformance LC (UPLC) system coupled to a quadrupole/time-of-flight MS system (TripleTOF 6600; SCIEX). Lipids were separated on an ACQUITY UPLC Peptide BEH C18 column (50 × 2.1 mm, 1.7 μm; Waters) using mobile phase (A) 1:1:3 (*v/v/v*) acetonitrile:methanol:water, and (B) 100% isopropanol. Both solvents were supplemented with 5 mM ammonium acetate and 10 nM ethylenediaminetetraacetic acid. The column was maintained at 45°C and a flow rate of 0.3 mL/min. A sample volume of 1 μL was used for injection. The gradient method was as follows: 0 min 0% (B); 1 min 0% (B); 5 min 40% (B); 7.5 min 64% (B); 12 min 64% (B); 12.5 min 82.5% (B); 19 min 85% (B); 20 min 95% (B); 20.1 min 0% (B); and 25 min 0% (B). The sample temperature was maintained at 4°C. Lipidomics data were analyzed using MS-DIAL (5.1.231129-dev).^39^ MS/MS spectra were visualized using Python (version 3.11) with code generated with assistance from ChatGPT with GPT-5 (OpenAI).

### Quantification and statistical analysis

Quantitative data are presented as mean ± standard deviation (SD), unless otherwise indicated. The mean was used as the measure of center, and SD was used as the measure of dispersion. The number of independent biological replicates (n) and the definition of n are indicated in the corresponding figure legends. For comparisons between two groups, Welch’s *t* test was used. For analyses involving two factors, two-way analysis of variance (ANOVA) followed by Tukey’s multiple-comparisons test was used, as indicated in the corresponding figure legends. For Figure 3, microbiota status (SPF versus GF) and tissue were used as factors. For Figure 4, age and tissue were used as factors. For Figures S3 and S4, microbiota status and age were used as factors. Statistical analyses were performed using GraphPad Prism (version 10.3.1) and Python (version 3.11). A two-sided *p* value <0.05 was considered statistically significant. Statistical significance is indicated as ∗*p* < 0.05, ∗∗*p* < 0.01, ∗∗∗*p* < 0.001, and ∗∗∗∗*p* < 0.0001; ns, not significant; ND, not detected.
