# Supplementary material for: Microbiome-dependent phytosterol sulfates production and tissue distribution in mice
Source: iScience. 2026 Oct 16;29(11):117883. doi: 10.1016/j.isci.2026.117883 (PMC13696130; doi:10.1016/j.isci.2026.117883)
Supplement: Supplementary file 1 [file mmc1.pdf]

**iScience, Volume *29***

## Supplemental information

# Microbiome-dependent phytosterol sulfates production and tissue distribution in mice

**Koki Tomita, Takeru Nakano, Satoshi Morozumi, Nobuyuki Okahashi, and Makoto Arita**

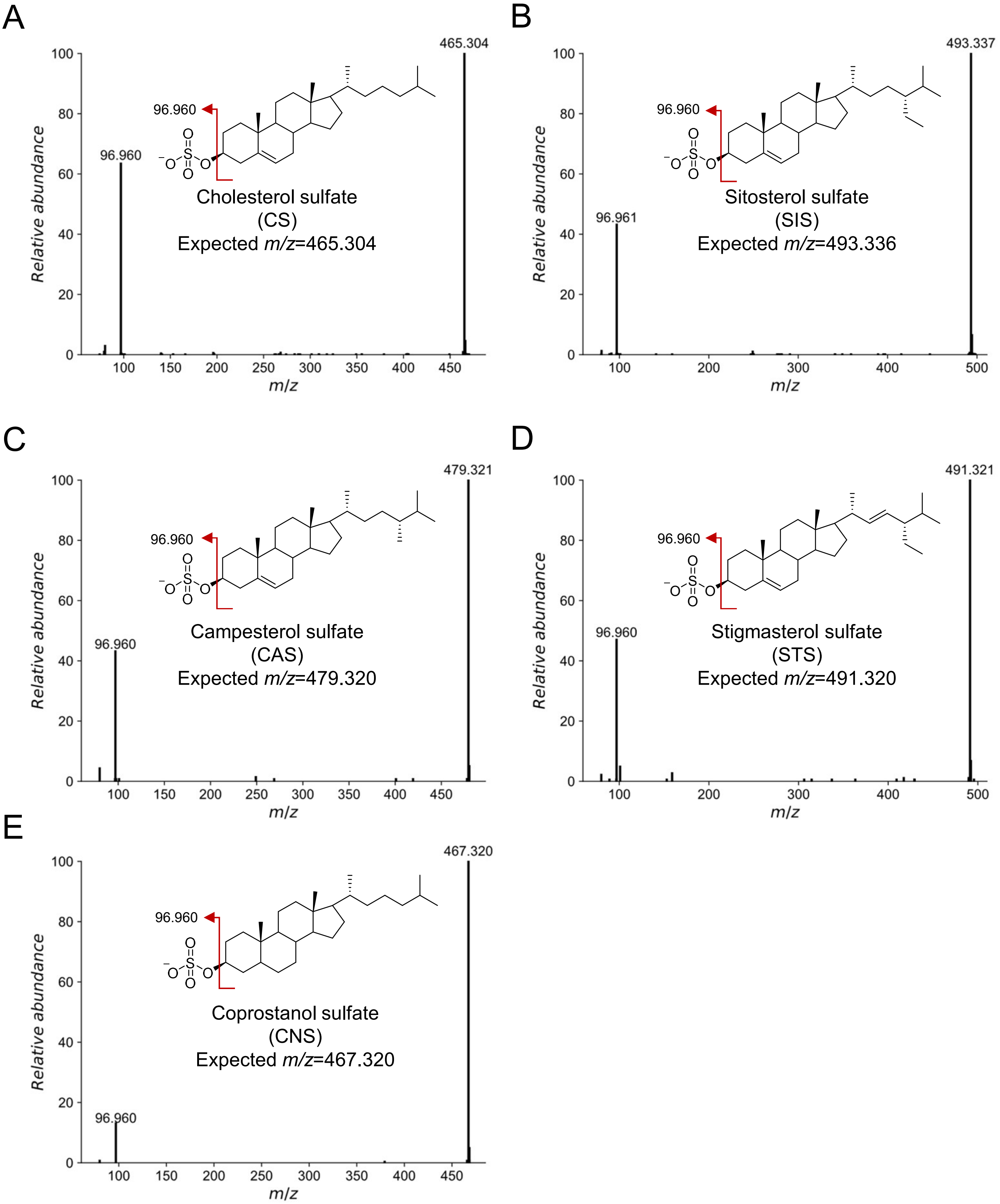


**Figure S1. MS/MS spectra of sterol sulfates in mouse feces.**
CS, cholesterol sulfate; SIS, sitosterol sulfate; CAS, campesterol sulfate; STS, stigmasterol sulfate; CNS, coprostanol sulfate.

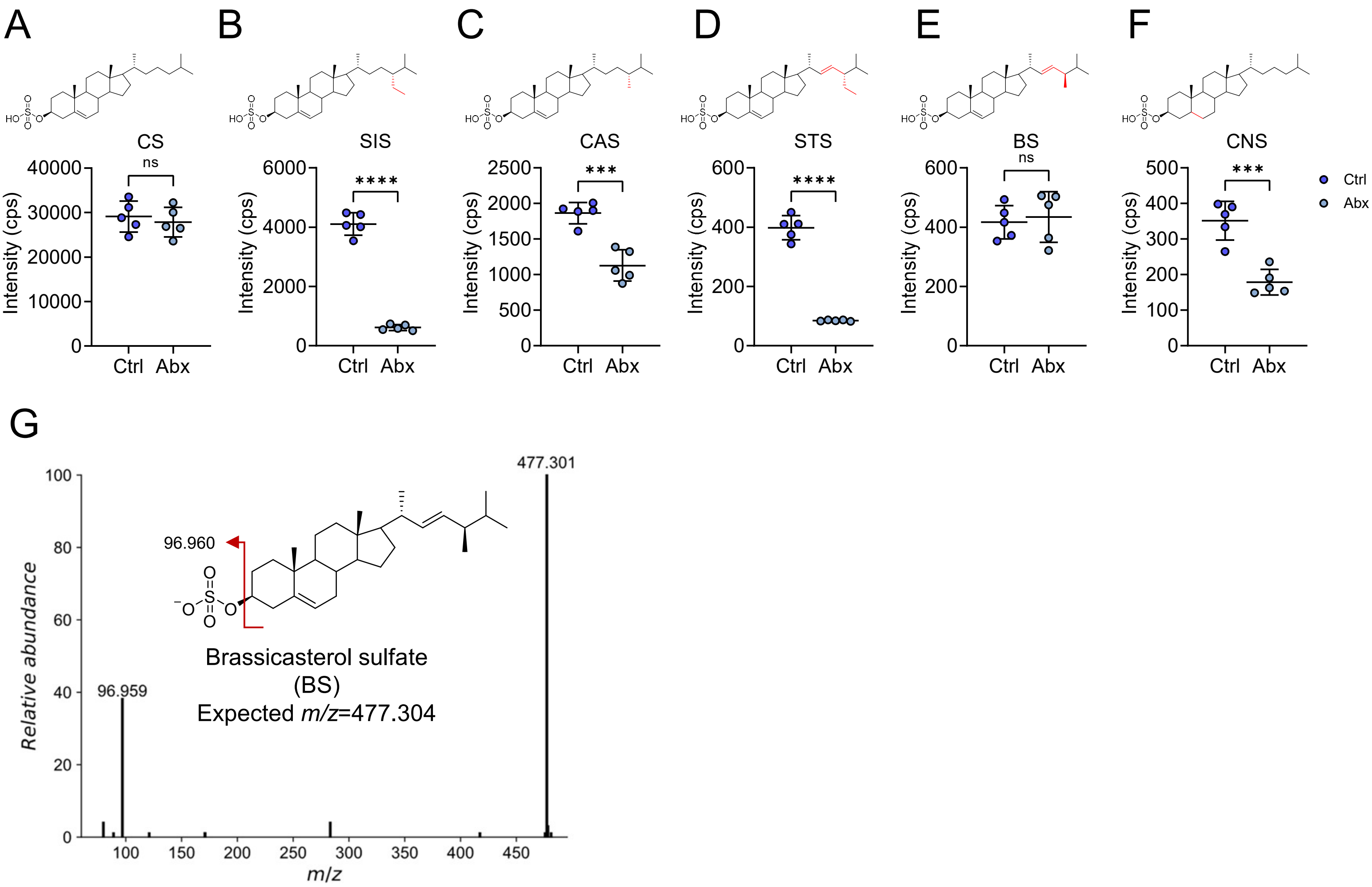


**Figure S2. Several sterol sulfates are reduced in feces of Abx-treated mice.**
(A-F) The raw intensities of sterol sulfates in mouse feces. Data are presented as mean ± SD. Each data point represents an individual mouse (n = 5 control mice and n = 5 antibiotic-treated mice). ***$p < 0.001$ and ****$p < 0.0001$; ns, not significant. Statistical significance was assessed using Welch's t-test. (G) MS/MS spectra of brassicasterol sulfate (BS) in mouse feces. CS, cholesterol sulfate; SIS, sitosterol sulfate; CAS, campesterol sulfate; STS, stigmasterol sulfate; CNS, coprostanol sulfate.

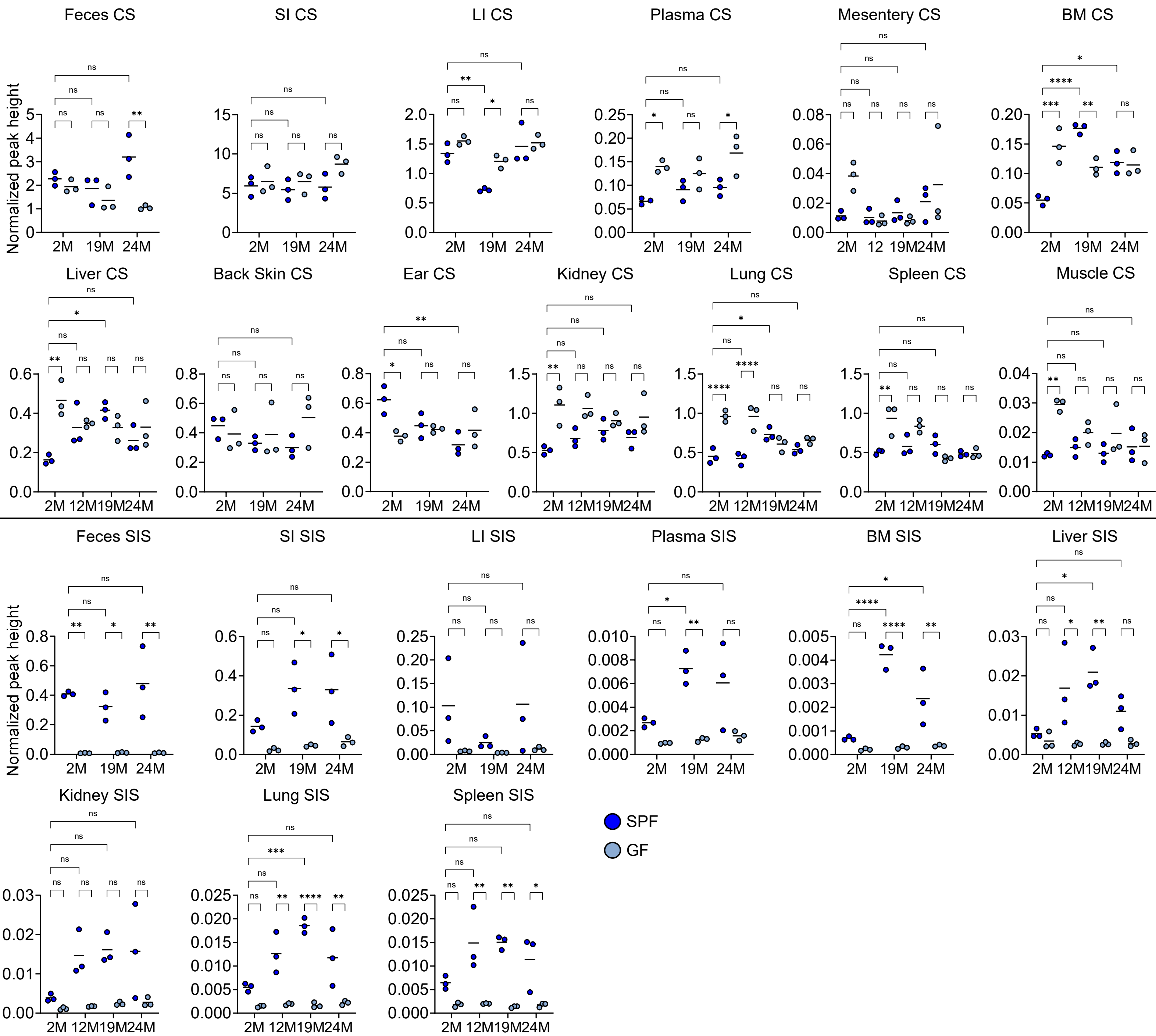


**Figure S3. Age- and bacteria-dependent distribution of cholesterol sulfate and sitosterol sulfate.**

Normalization was performed by dividing the raw intensities of sterol sulfates by the raw intensity of d7-lysophosphatidylcholine added as an internal standard. Data are presented as mean ± SD. Each data point represents an individual mouse (n = 3 per group). $^{*}p < 0.05$, $^{**}p < 0.01$, $^{***}p < 0.001$ and $^{****}p < 0.0001$; ns, not significant. Statistical significance was assessed using two-way ANOVA with microbiota status and age as factors, followed by Tukey's multiple-comparisons test. CS, cholesterol sulfate; SIS, sitosterol sulfate.

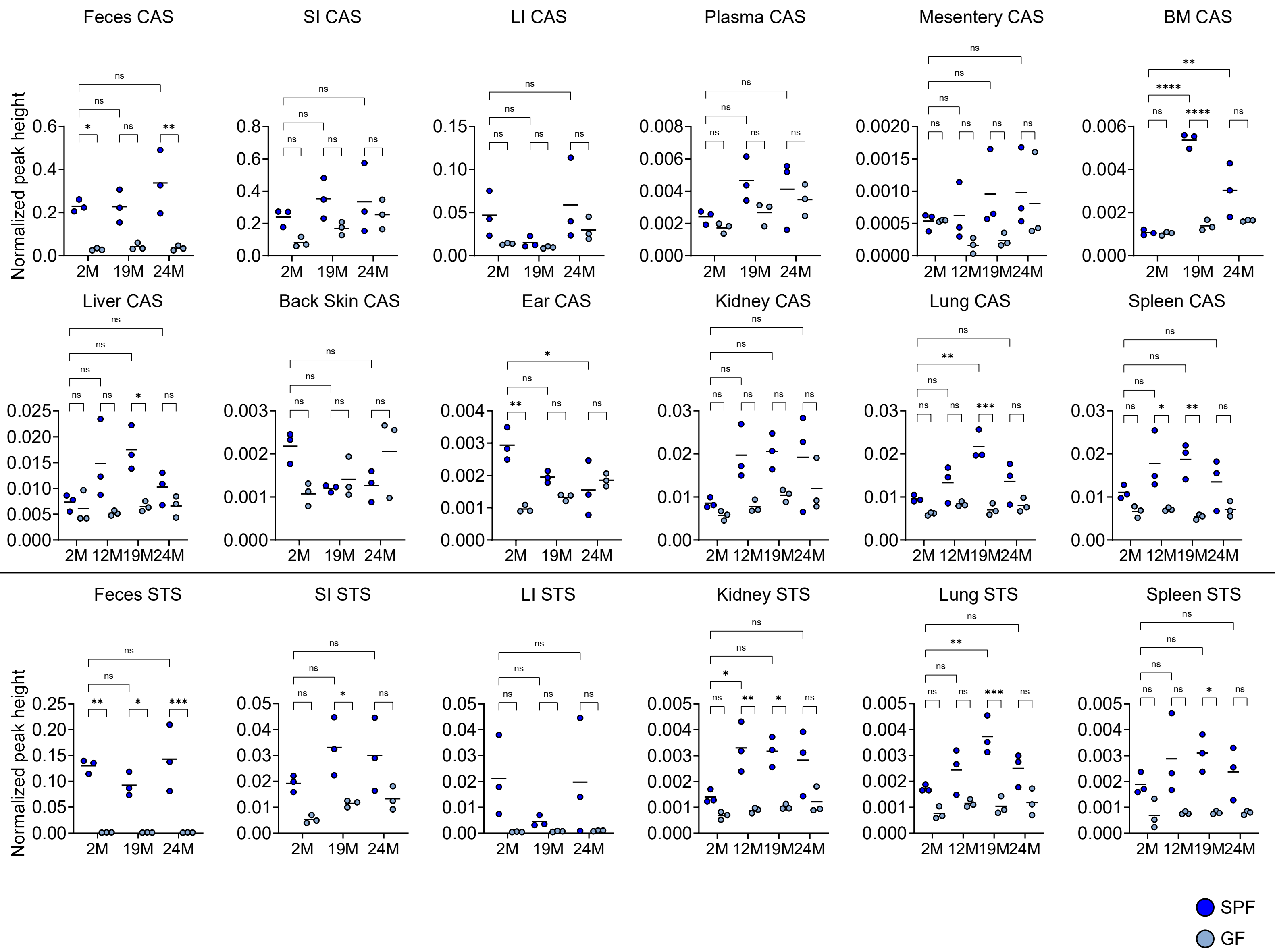


**Figure S4. Age- and bacteria-dependent distribution of campesterol sulfate and stigmasterol sulfate.**

Normalization was performed by dividing the raw intensities of sterol sulfates by the raw intensity of d7-lysophosphatidylcholine added as an internal standard. Data are presented as mean ± SD. Each data point represents an individual mouse (n = 3 per group). *$p < 0.05$, **$p < 0.01$, ***$p < 0.001$ and ****$p < 0.0001$; ns, not significant. Statistical significance was assessed using two-way ANOVA with microbiota status and age as factors, followed by Tukey's multiple-comparisons test. CAS, campesterol sulfate; STS, stigmasterol sulfate.

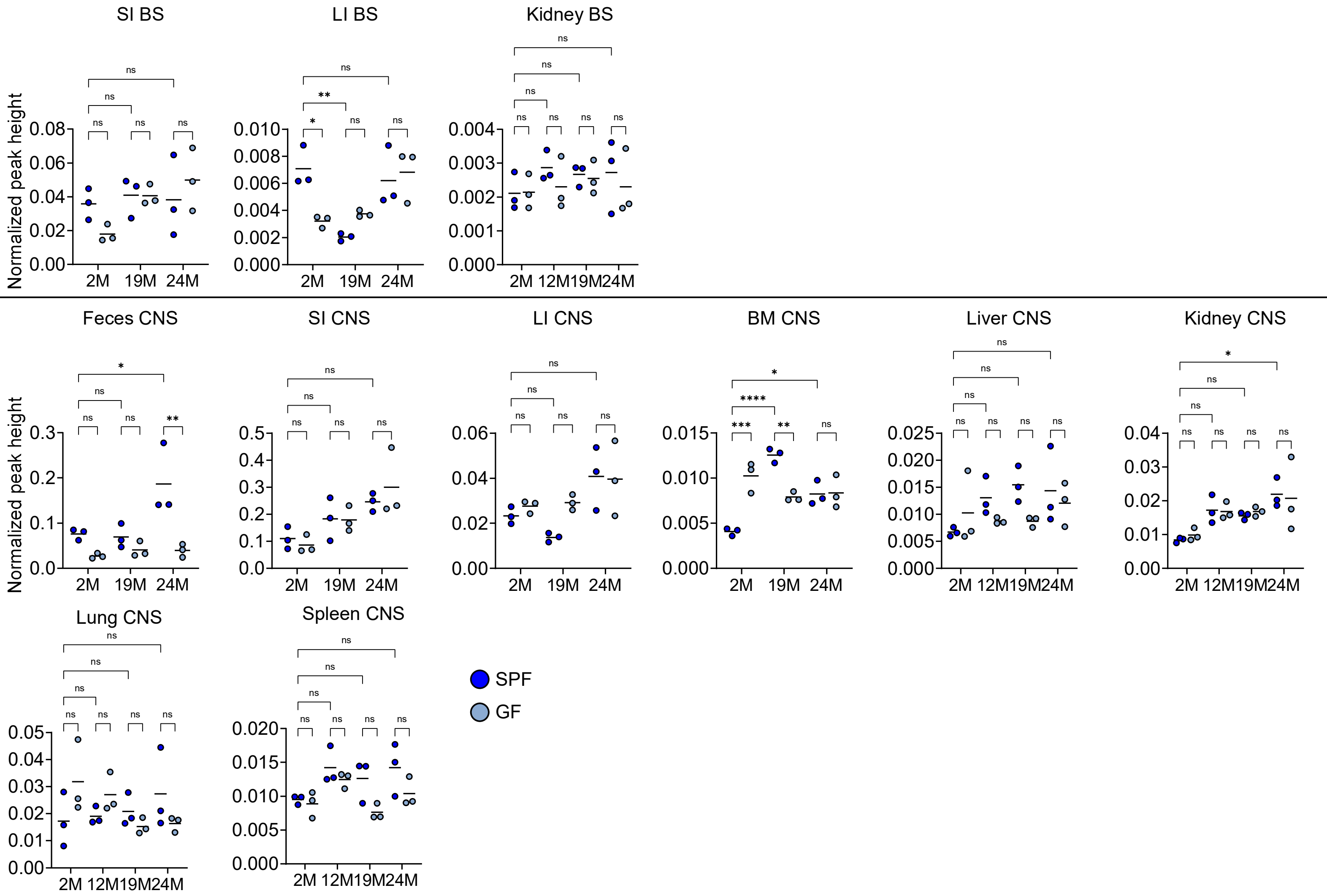


**Figure S5. Age- and bacteria-dependent distribution of brassicasterol sulfate and coprostanol sulfate.**

Normalization was performed by dividing the raw intensities of sterol sulfates by the raw intensity of d7-lysophosphatidylcholine added as an internal standard. Data are presented as mean ± SD. Each data point represents an individual mouse (n = 3 per group). *$p < 0.05$, **$p < 0.01$, ***$p < 0.001$ and ****$p < 0.0001$; ns, not significant. Statistical significance was assessed using two-way ANOVA with microbiota status and age as factors, followed by Tukey's multiple-comparisons test. BS, brassicasterol sulfate; CNS, coprostanol sulfate.

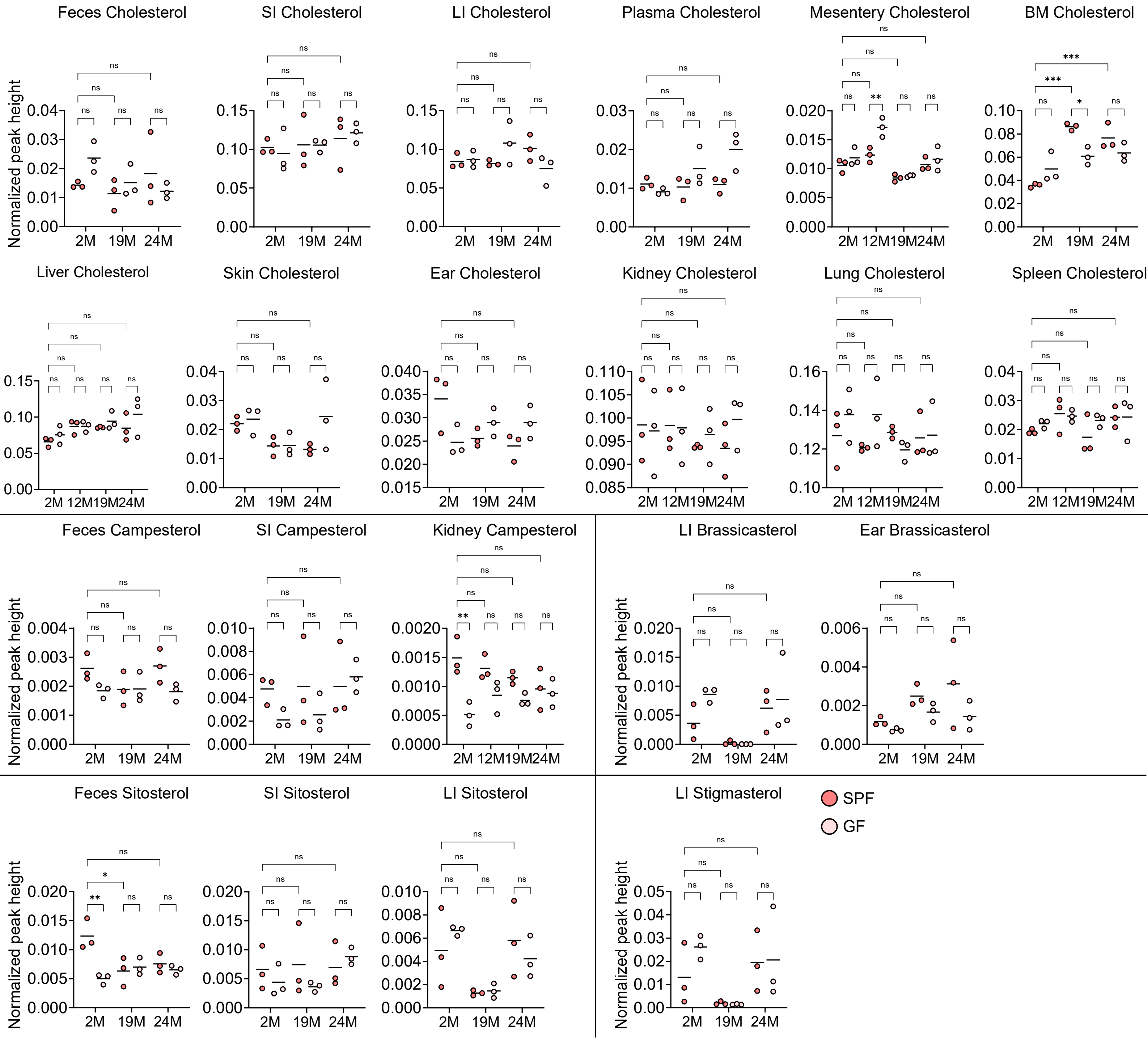


**Figure S6. Age- and bacteria-dependent distribution of sterols.**
Normalization was performed by dividing the raw intensities of sterols by the raw intensity of d7-monoacylglycerols added as an internal standard. Data are presented as mean ± SD. Each data point represents an individual mouse (n = 3 per group). *$p$ < 0.05, **$p$ < 0.01 and ***$p$ < 0.001; ns, not significant. Statistical significance was assessed using two-way ANOVA with microbiota status and age as factors, followed by Tukey's multiple-comparisons test.

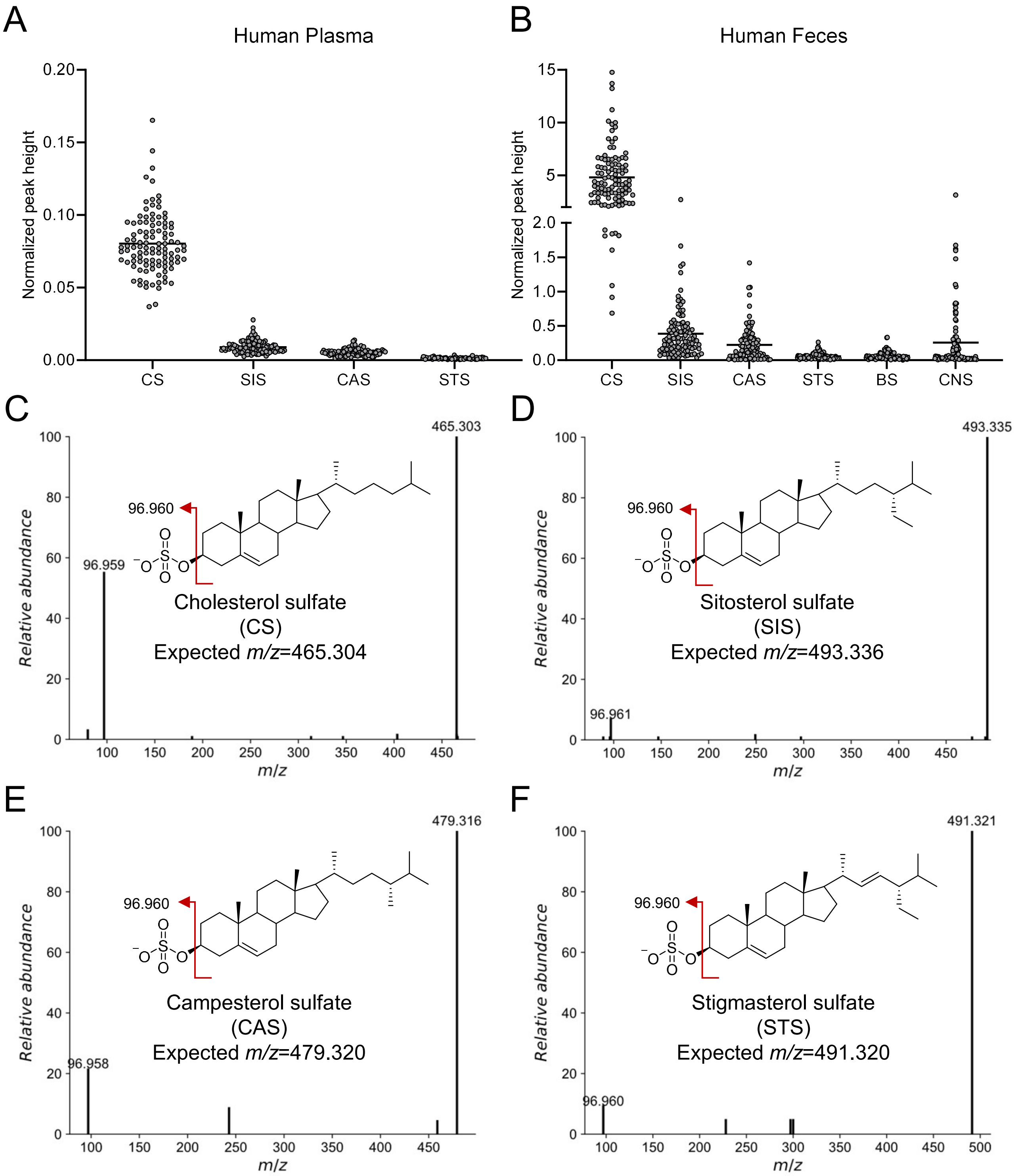


**Figure S7. Sterol sulfates detected in human plasma and feces.**

(A, B) The normalized intensities of sterol sulfates detected in healthy control plasma (A) and feces (B). Normalization was performed by dividing the raw intensities of sterol sulfates by the raw intensity of d7-lysophosphatidylcholine added as an internal standard. Data are presented as mean values from 107 healthy participants (n = 107). (C-F) MS/MS spectra of CS (C), SIS (D), CAS (E), STS (F) detected in human plasma. CS, cholesterol sulfate; SIS, sitosterol sulfate; CAS, campesterol sulfate; STS, stigmasterol sulfate; BS, brassicasterol sulfate; CNS, coprostanol sulfate.

| Primer sequence | Sequence (5'-3') |
|---|---|
| Forward primer to amplify *sult* of *Bacteroides thetaiotaomicron* | GAAGGAGATATACATATGGGATTACTTGAATTTAATAAAC |
| Reverse primer to amplify *sult* of *Bacteroides thetaiotaomicron* | GTGGTGGTGCTCGAGTAAATTATAGTTCCATTGTTTCAAA |
| Forward primer to amplify *sult* of *Bacteroides fragilis* | GAAGGAGATATACATATGGGATTACTTGAATTTAACAAAC |
| Reverse primer to amplify *sult* of *Bacteroides fragilis* | GTGGTGGTGCTCGAGTAAACTATAATCCCATTGTTTCAGT |
| Forward primer to amplify *sult* of *Bacteroides uniformis* | GAAGGAGATATACATATGGGATTGTTAGAATTCAATAAGC |
| Reverse primer to amplify *sult* of *Bacteroides uniformis* | GTGGTGGTGCTCGAGTAAATCGTACTTCCACTGTTCCAGC |
| Forward primer to amplify *sult* of *Bacteroides caccae* | GAAGGAGATATACATATGGGATTACTCGAATTTAATAAAC |
| Reverse primer to amplify *sult* of *Bacteroides caccae* | GTGGTGGTGCTCGAGTAAATTATAATCCCATTGTTTCAAA |
| Forward primer to amplify *sult* of *Parabacteroides goldsteinii* | GAAGGAGATATACATATGGGGTTGCTGGAATTTGATAAAT |
| Reverse primer to amplify *sult* of *Parabacteroides goldsteinii* | GTGGTGGTGCTCGAGTAAAGAATATCCCCACTCCTTGAGA |
| Forward primer to amplify *sult* of *Parabacteroides johnsonii* | GAAGGAGATATACATATGGGATTACTGGAATTTGACAAAC |
| Reverse primer to amplify *sult* of *Parabacteroides johnsonii* | GTGGTGGTGCTCGAGTAAAGAATATCCCCACTCCTTGAGA |
| Forward primer to amplify mouse *Sult2b1* | GAAGGAGATATACATATGGACGGCCCGCAGCCGCG |
| Reverse primer to amplify mouse *Sult2b1* | GTGGTGGTGCTCGAGCTGGGATGAACCCGGATTCGG |
| Forward primer to amplify human *SULT2B1* | GAAGGAGATATACATATGGATGGCCCGGCGGAACCGCAAA |
| Reverse primer to amplify human *SULT2B1* | GTGGTGGTGCTCGAGCGATGGGCGCGGATG |

**Table S1. Oligonucleotides used for cloning *sult* genes into pET21a(+) vector**
All oligonucleotides in this table were purchased from Sigma-Aldrich.
